# Correlation between Vitamin D levels and thyroid autoantibodies in newly diagnosed hashimoto's thyroiditis patients

**DOI:** 10.4314/ahs.v25i2.22

**Published:** 2025-06

**Authors:** Ahmed Cihad Genç, Aysel Toçoğlu, Fatma Betül Asan, Attila Önmez

**Affiliations:** 1 Sakarya University Training and Research Hospital, Department of Internal Medicine, Sakarya, Turkey; 2 Duzce University, Department of Internal Medicine, Duzce, Turkey

**Keywords:** Hashimoto's Thyroiditis, Thyroglobulin Antibody, Thyroid Peroxidase Antibody, Vitamin D

## Abstract

**Background:**

Vitamin D plays a role in numerous metabolic functions. In this study we aimed to assess vitamin D levels in Hashimoto's Thyroditis (HT) patients, and demonstrate the relationship between these levels and thyroid function tests or thyroid autoantibodies.

**Methods:**

A retrospective analysis of 112 newly diagnosed euthyroid HT (EHT) patients and 178 healthy controls was carried out. 25-OH D level was classified as vitamin D deficiency, vitamin D insufficiency, and vitamin D sufficient.

**Results:**

Mean age was 35.33±11.54 years in the patient group, and 35.84±10.9 in the control group (p=0.777). Compared with the healthy controls, patients in the EHT group had significantly lower vitamin D levels (p=0.007), and a significantly higher prevalence of vitamin D deficiency (p=0.001). While there was a negative correlation between vitamin D levels and anti-TPO values (r=-0.133, p=0.023), there was no significant relationship between vitamin D and TSH, fT4, fT3, and anti-TG values (p>0.05).

**Conclusıons:**

This study showed that EHT patients had lower vitamin D levels compared to healthy controls, and there was a significant negative correlation between vitamin D levels and anti-TPO levels. Based on our study, patients diagnosed with HT should undergo vitamin D screening, and replacement if necessary.

## Introductıon

Hashimoto's thyroditis is a chronic autoimmune disease characterized by destruction of thyroid tissue, caused by lymphocytic infiltration. It has an annual incidence of 0.3-1.5 per 1000, and is the most common cause of hypothyroidism in areas of iodine sufficiency^1^,^2^. It is especially common in young and middle aged females, with a female to male ratio of 7/1 2. Although studies have stated that the disease is triggered by environmental factors in the presence of a genetic tendency, the etiology remains uncertain^3^. The most commonly known risk factors include iodine excess, viral infections, chemicals, and drugs that stimulate antibody development such as amiodarone^2^. Recent studies focused on the efects of dietary factors, including deficiencies of vitamin D and selenium^4^. Because patients in early disease stages are generally euthyroid, they are asymptomatic and the diagnosis is made incidentally during routine screening. During the course, sublinical hypothyroidism, overt hypothroidism with or without goiter, or even thyroid cancer may develop sequentially^5^. It is therefore important to detect and monitor the disease in the healthy population, and manage hypothyroidism.

Vitamin D is a secosteroid structured molecule produced in the skin. In addition to its fundamental role in bone mineralization and calcium metabolism, it also has important modulating effects on the immune system, exerted through the vitamin D receptor (VDR)^6^. Immunomodulator effects are exerted through VDR. Activation of VDR in immune cells leads to the transcription of gene products that trigger the antiproliferative and immune regulatory cascade^6^. Numerous studies have suggested that vitamin D deficiency is associated with autoimmune diseases, metabolic syndrome, chronic inflammatory disorders, cardiovascular diseases, and cancer^7^-^10^. Despite numerous recent studies on the role of vitamin D in autoimmune thyroid diseases, the results are controversial^11^,^12^. There are also studies which suggest that vitamin D supplementation has a protective role in autoimmune thyroid diseases and thyroid cancers^13^.

The aim of this study is to analyze vitamin D levels in euthyroid Hashimoto's thyroiditis (EHT) patients and to demonstrate the relationship between vitamin D levels, thyroid function tests, and autoantibodies.

## Methods

This study included 112 patients who presented to the internal medicine clinics of our hospital between 01.03.2023 and 01.06.2023, and who were diagnosed with HT by laboratory tests. The study also included 178 healthy controls who presented for a health screening during the same time period. All participants were females between ages 18-65 years. Patients who smoked or consumed alcohol, had comorbidities, a history of thyroidectomy or pregnancy, hormone replacement prior to presentation, use of anti thyroid or drugs that could affect thyroid function (e.g. amiodarone, lithium), and a history of vitamin D or calcium replacement during the previous 6 months were not included. The study was carried out according to the declaration of Helsinki. All procedures in the study were approved by the Duzce University Ethical Board (Ethical board number: 2023/149). Informed consents were not taken because the study was retrospective.

### Demographic and Laboratory Data

Subject data including age, sex, laboratory studies, and thyroid ultrasound results were retrieved from clinic files and retrospectively recorded on the hospital information system. Height and weight measurements on the hospital records were used to calculate the body mass index (BMI) (kg/m2). Blood samples were taken after minimum 8 hours of fasting. Fasting blood glucose (FBG), urea, creatinine, aspartate aminotransferase (AST), alanine aminotransferase (ALT) were assessed with Beckman Coulter AU5800 device, free tetraiodothyronine (fT4), free triiodothyronine (fT3), thyroid stimulating hormone (TSH), thyroid peroxidase antibody (anti-TPO), thyroglobulin antibody (Anti-TG), and 25-Hydroxyvitamin D (25-OH D) were assessed with Siemens Advia Centaur XPT device.

### Diagnostic Criteria

HT was diagnosed with a positive thyroid antibody test (anti-TPO>60 IU/mL and/or anti-TG>4.5 IU/mL) and presence of diffuse heterogeneity on ultrasound consistent with chronic thyroiditis. Euthyroidism was defined with TSH (0.35-5.5 mIU/L) and fT4 (0.83–1.43 ng/dL) levels within the normal reference range. Based on previous studies and guidelines, a 25-OH D level <20 µg/L was classified as vitamin D deficiency, 20-29.9 µg/L as vitamin D insufficiency, and ≥30 µg/L as sufficient vitamin D^14^,^15^.

### Statistical Analysis

Descriptive analyses were carried out to provide comprehensive insights into the fundamental characteristics of the study cohort. Both visual representations, encompassing probability plots and histograms, along with rigorous analytical techniques such as the Kolmogorov-Smirnov and Shapiro-Wilk's tests, were employed to detect the normal distribution status of the data. Parametric assessments, especially the Independent Sample t-test, were applied to compare these parameters. The examination of categorical variables between the two distinct groups was made by the chi-square test. The categorical variables were shown in terms of frequency percentages. For the investigation of data with parametric attributes involving multiple samples and repeated measures, a Repeated Measures Variance Analysis (ANOVA) was made. In this context, the Greenhouse-Geisser correction method was used. Given the normal distribution of both variables, the Pearson correlation coefficient in conjunction with its associated significance levels was calculated. The correlation analysis was also separately conducted for distinct work status categories. p<0.05 was set for statistical significance. All analytical procedures were executed utilizing the SPSS statistical software (IBM SPSS Statistics, Version 22.0. Armonk, NY: IBM Corp.).

## Results

A total of 290 women, including 112 (38.6%) EHT patients and 178 (61.4%) healthy controls, were included into the study. The demographic, anthropometric, and laboratory parameters of the EHT and healthy control groups are summarized in Table I. Mean age was 35.33±11.54 in the EHT group, and 35.84±10.95 in the healthy control group (p=0.777). Evaluation of the biochemical parameters showed that the EHT group had significantly higher levels of TSH, anti-TPO, and anti-TG levels (p<0.001), and lower fT4 and vitamin D levels compared to the healthy group (p<0.05). Mean vitamin D level was 11.43±5.19 µg/L in the EHT group, and 14.53±8.25 µg/L in the healthy group. Classification of subjects in both groups with respect to vitamin D levels showed that the ratio of patients with vitamin D deficiency was significantly higher in the EHT than the healthy group (90.2% vs. 74.7%, respectively) (p=0.001). While there were 13 patients with normal vitamin D levels in the healthy group, there were no patients in the EHT group who had sufficient vitamin D levels.

**Table I:** Demographic, anthropometric, and laboratory parameters of EHT and healthy groups

	Total (n=290)	Healthy Group (n=178)	EHT Group (n=112)	p
**Age, years**	35.03±10.17	35.84±10.95	35.33±11.54	0.777
**Sex, female, n(%)**	290 (100)	178 (100)	112 (100)	-
**BMI, kg/m^2^**	26.30±6.18	25.65±5.27	27.35±7.34	0.244
**Glucose, mg/dL**	90.56±8.08	90.35±8.05	90.89±8.16	0.945
**Urea, mg/dL**	23.15±6.87	23.44±7.36	22.69±6.01	0.572
**Creatinine, mg/dL**	0.61±0.10	0.62±0.10	0.60±0.11	0.159
**AST, U/L**	17.74±5.49	17.83±5.17	17.60±5.99	0.366
**ALT, U/L**	15.77±9.72	15.43±8.30	16.29±1.64	0.965
**fT4, ng/dL**	1.07±0.15	1.09±0.16	1.05±0.13	**0.022**
**fT3, ng/L**	3.09±0.15	3.07±0.52	3.13±0.49	0.387
**TSH, mlU/L**	1.99±1.02	1.78±0.88	2.33±1.14	**<0.001**
**Anti TPO, IU/mL**	234.23±432.61	15.22±15.72	582.30±536.38	**<0.001**
**Anti TG, IU/mL**	36.35±128.57	1.82±1.34	93.23±195.16	**<0.001**
**25-OH D, µg/L**	13.33±7.37	14.53±8.25	11.43±5.19	**0.007**
**Patient prevalence, n(%)**				
25-OH D<20 µg/L*	234 (80.7)	133 (74.7)	101 (90.2)	
**25-OH D:20-29.9 µg/L**	43 (14.8)	32 (18.0)	11 (9.8)	**0.001**
25-OH D>30 µg/L*	13 (4.5)	13 (7.3)	0 (0.0)	

*: There is a statistically significant difference between the groups. Abbreviations: EHT= Euthyroid Hashimoto's Thyroiditis, BMI= body mass index; AST= Aspartate aminotransferase; ALT= Alanine aminotransferase; fT4= free tetraiodothyronine; fT3= free triiodothyronine; TSH= thyroid stimulating hormone; Anti-TPO= thyroid peroxidase antibody; Anti-TG= thyroglobulin antibody; 25-OH D= 25-Hydroxy Vitamin D

The subjects were separated into 3 groups with respect to vitamin D levels: 234 (80.7%) had vitamin D deficiency, 43 (14.8%) had vitamin D insufficiency, and 13 (4.5%) had adequate levels of vitamin D. The demographic, anthropometric, and laboratory parameters of the 3 groups are summarized in Table II. Patients with vitamin D deficiency were significantly younger (p=0.01). The levels of anti-TPO, anti-TG and the number of patients positive for anti-TPO were significantly higher in the groups with vitamin D deficiency or insufficiency, compared to the group with sufficient vitamin D (p<0.001, p=0.026, p=0.006, respectively). There were no significant differences between the 3 groups with respect to TSH, fT4, fT3 levels and the number of anti-TG positive patients (p>0.05).

**Table II:** Demographic, anthropometric, and laboratory parameters of subjects with vitamin D deficiency, insufficiency, and with sufficient vitamin D

	Group with vitamin D deficiency (n=234)	Group with vitamin D insufficiency (n=43)	Group with sufficient vitamin D (n=13)	p
**Age, years**	34±10	37±11	41±7	**0.010**
**Sex, Female, n(%)**	234 (100)	43 (100)	13 (100)	-
**BMI, kg/m^2^**	26.17±6.27	27.02±6.34	26.31±4.63	0.709
**Glucose, mg/dL**	90.21±8.00	91.79±8.42	92.77±8.32	0.138
**Urea, mg/dL**	23.35±7.10	21.60±4.92	24.69±7.73	0.270
**Creatinine, mg/dL**	0.61±0.11	0.62±0.07	0.67±0.13	0.255
**AST, U/L**	17.88±5.81	16.88±3.67	17.92±4.25	0.543
**ALT, U/L**	15.91±10.50	15.33±5.70	14.62±4.17	0.877
**fT4, ng/dL**	1.08±0.14	1.07±0.18	1.05±0.16	0.652
**fT3, ng/L**	3.09±0.50	3.10±0.55	3.01±0.16	0.859
**TSH, mlU/L**	2.02±1.02	1.90±1.02	1.70±1.13	0.155
**Anti TPO, IU/mL**	251.89±442.37	206.69±428.58	7.32±10.63	**<0.001**
**Anti TG, IU/mL**	39.00±136.80	32.38±97.56	1.83±1.77	**0.026**
**Patient prevalence**				
Anti TPO > 60 IU/mL	91 (38.9)	11 (25.6)	0 (0.0)	**0.006**
Anti TG > 4.5 IU/mL	64 (27.4)	9 (20.9)	1 (7.7)	0.216

Abbreviations : BMI= Body mass index, AST= Aspartate aminotransferase; ALT= Alanine aminotransferase; fT4= free tetraiodotyronine; fT3= free triiodothyronine; TSH= thyroid stimulating hormone; Anti-TPO= thyroid peroxidase antibody; Anti-TG= thyroglobulin antibody; 25-OH D= 25-Hydroxy Vitamin D

The correlation analysis made to determine the association of vitamin D levels with thyroid autoantibodies and thyroid function tests showed a statistically significant negative correlation between vitamin D level and anti-TPO level(r=-0.133, p=0.023). However there was no significant relationship between vitamin D levels and TSH, fT4, fT3, and anti-TG levels (r=-0.093, p=0.113; r=-0.093, p=0.113; r=0.04, p=0.435 and r=-0.109, p=0.063, respectively) (Table III).

**Table III:** Correlation analysis between TSH, fT4, fT3, anti-TPO, anti-TG, and 25-OH D vitamin levels (Pearson Coefficient)

n=290		TSH	fT4	fT3	Anti-TPO	Anti-TG	25-OH D
**TSH**	**r**	1.000	-0.071	0.064	0.296	0.169	-0.093
	**p**		0.226	0.281	**0.000**	**0.004**	0.113
**fT4**	**r**		1.000	0.079	-0.017	-0.065	0.036
	**p**			0.180	0.769	0.270	0.546
**fT3**	**r**			1.000	0.118	0.086	0.046
	**p**				0.044	0.142	0.435
**Anti-TPO**	**r**				1.000	0.518	-0.133
	**p**					**0.000**	**0.023**
**Anti-TG**	**r**					1.000	-0.109
	**p**						0.063

Abbreviations: TSH= thyroid stimulating hormone; fT4= free tetraiodothyronine; fT3= free triiodothyronine; Anti-TPO= thyroid peroxidase antibody; Anti-TG= thyroglobulin antibody; 25-OH D= 25-Hydroxy Vitamin D

The odds ratio was calculated for risk analysis when the cut-off value for vitamin D was taken as 20. In the presence of vitamin D deficiency there was a 3.107 fold increase in HT (%95 CI: 1.530–6.307, p= 0.001), and 2.604 fold increase in anti-TPO positivity (%95 CI: 1.280–5.291, p= 0.004).

## Dıscussıon

Hashimoto's thyroiditis is the most common autoimmune thyroid disorder, and its incidence has shown an increase in recent years. During the course of the disease, patients may develop hypothyroidism, lymphoma possibly related to HT, and papillary thyroid cancer. An effective treatment that can prevent these has not been found 5. Based on the data acquired in the last 30 years, it is widely accepted that vitamin D plays a critical role in the modulation of the immune system. Although the pathogenesis of autoimmune thyroid diseases is not known completely, studies have shown a possible association between vitamin D deficiency and initiation or progression of the diseases^16^. In our study we aimed to show the relationship between HT and vitamin D, and determine the correlation between thyroid function tests and autoantibodies with vitamin D levels.

The study included 290 women, 112 (38.6%) were EHT patients, and 178 (61.4%) were healthy controls. Mean age in the EHT group was 35±11 years, and was consistent with previous reports. Mean vitamin D level was 11.43±5.19 µg/L in the EHT group, and 14.53±8.25 in the healthy control group. Vitamin D level in the ETH group was significantly lower compared to the healthy control group (p<0.05). Also, when the subjects in both groups were classified according to vitamin D levels, the number of vitamin D deficient patients was significantly higher in the EHT group (90.2%) compared to the healthy group (74.7%) (p=0.001). Similar to ours, previous studies found that HT patients had significantly lower levels of vitamin D and a higher prevalence of vitamin Deficiency compared to healthy controls^17^,^18^. Another study emphasized that there was a relationship between the severity of vitamin D and duration of HT, thyroid volume, and antibody levels. Also, vitamin D level had a potential role in the onset or progression of HT^16^. Contradictory to these data, a contemporary study published in 2021 and included 456 HT patients reported that there was no difference between HT patients and healthy controls with respect to vitamin D levels and prevalence of vitamin D deficiency^4^.

While the prevalence of vitamin D deficiency is investigated in HT patients, the prevalence of HT in patients with vitamin D deficiency was also studied. Studies showed that the incidence of HT is increased in patients with vitamin D deficiency, compared to those with normal vitamin D levels^19^,^20^. Prospective studies in which vitamin D replacement was given showed that anti-TPO and/or anti TG levels decreased significantly after vitamin D replacement. Ucan et al found that in EHT patients, vitamin D replacement led to a statistically significant decrease in antibody levels and thyroid volüme^21^. In another study by Krysiak et al, the subjects received 2000 IU/day vitamin D replacement for 6 months^22^. At the end of this period serum vitamin D levels increased significantly, both anti-TPO and anti-TG levels decreased significantly with greater decreases in anti-TPO levels^22^. In our study, 80.7% of the patients had vitamin D deficiency and 14.8% had vitamin D insufficiency. Similar to previous tudies, comparison of the number of antibody positive patients and antibody titers between the groups showed that the anti-TPO and anti-TG levels and the number of anti-TPO positive patients were significantly higher in the groups with deficiency or insufficiency of vitamin D, compared to the group with sufficient vitamin D (p<0.001, p=0.026, p=0.006, respectively). This showed us that there was a negative relationship between vitamin D levels and antibody levels.

Correlation analysis for determining the relationship between vitamin D and thyroid autoantibodies revealed statistically significant negative correlation between vitamin D levels and anti-TPO levels. (r=-0.133, p=0.023). Shin et al compared 111 antibody positive patients with 193 antibody negative patients, and found that levels of vitamin D were significantly lower in antibody positive patients^23^. In consistence with our study they also found a negative correlation between vitamin D and anti-TPO levels (r=-0.252; p<0.001)^23^. In some other important studies a negative correlation between vitamin D and anti-TG levels, however we did not find such a relationship^20^,^24^. Studies evaluating the relationship between vitamin D and thyroid function tests showed that vitamin D level is associated with TSH, the higher the level of vitamin D the lower was the TSH level^12^,^25^. Chao et al found that with every 1ng/mL increase in vitamin D there was a 0.17 mIU/L decrease in TSH level, and they stated that vitamin D supplementation could play a role in preventing hypothyroidism^15^. However, this relationship was not confirmed by other studies. A meta analysis that was published in 2021 and evaluated 6 clinical studies showed that vitamin D levels increased and anti-TPO titers decreased significantly with vitamin D replacement, however a relationship between vitamin D levels with thyroid function tests and anti-TG levels could not be found 26. Similarly, we did not find a significant correlation between vitamin D levels with either anti-TG levels or thyroid function tests. These findings show that anti-TPO is a better reflector of the effects of vitamin D on thyroid autoimmunity than the anti-TG.

The risk analysis in our study showed that in vitamin D deficiency, the presence of HT increased by 3.107 fold (OR: 3.107, %95 CI: 1.530-6.307, p= 0.001), and anti-TPO positivity increased by 2.604 fold (OR: 2.604, %95 CI: 1.280-5.291, p= 0.004). Mansournia et al showed that a 5 ng/ml increase in vitamin D level led to 19% decrease in odds of HT^27^.

Our study had some limitations. The retrospective and single center nature of the study, the relatively small number of EHT patients, and inclusion of only females were the major limitations. Although the lack of subclinical and overt hypothyroidism groups led to limitations in detecting the relationship between vitamin D and thyroid function tests, the inclusion of only newly diagnosed euthyroid patients rendered the patient group more specific. Another advantage of the study was its conduction in a single season to exclude seasonal factors.

## Conclusion

In conclusion, this study showed a higher prevalence of vitamin D deficiency in HT patients compared to healthy controls, and also a higher prevalence of HT in patients with either deficiency or insufficiency of vitamin D compared to individuals with normal vitamin D levels. While a negative correlation was found between vitamin D and anti-TPO levels, there was no significant relationship between vitamin D and TSH, fT4, fT3, and anti-TG values. More comprehensive and prospective studies are needed to clarify the relationship between thyroid autoimmunity and vitamin D, and to determine the role of vitamin D supplementation in the prevention and alleviation of thyroid autoimmunity. In the meantime, vitamin D screening should be carried out at the time of diagnosis or during follow up of patients diagnosed with HT, and replacement should be recommended if necessary.

## Data Availability

The data and materials generated/analyzed in the present study are available from the corresponding author upon request.
